# Serious infections in children: an incidence study in family practice

**DOI:** 10.1186/1471-2296-7-23

**Published:** 2006-03-28

**Authors:** Ann Van den Bruel, Stefaan Bartholomeeusen, Bert Aertgeerts, Carla Truyers, Frank Buntinx

**Affiliations:** 1 Academisch Centrum voor Huisartsgeneeskunde, Katholieke Universiteit Leuven, Leuven, Belgium; 2 Department of General Practice, Universiteit Maastricht, Maastricht, The Netherlands

## Abstract

**Background:**

Information on the incidence of serious infections in children in general practice is scarce. However, estimates on the incidence of disease are important for several reasons, for example to assess the burden of disease or as a basis of diagnostic research. We therefore estimated the incidence of serious infections in general practice in Belgium.

**Methods:**

Intego is a morbidity registration network, in which 51 general practitioners continuously register all diagnoses and additional data in their electronic medical records. Serious infections were defined as pneumonia, sepsis, meningitis, pyelonephritis and osteomyelitis. Incidences are calculated for the period of 1998 to 2002, per 1000 patients in the yearly contact group, which is the group of patients that consulted their GP at least once that year, and in the practice population, which is the estimated true population of that practice.

**Results:**

The incidence of all infectious diseases peaks in children between 0 and 4 years, with 1731 infections per 1000 children per year in the yearly contact group. Incidence drops with increasing age: 972 infections per 1000 children per year in children between 5 and 9 years old, and 732 in children between 10 and 14 years old. The same decline in incidence is observed in the subgroup of serious infections: 21 infections per 1000 children per year in children between 0 and 4 years, 12 in children between 5 and 9 years and 5 in children between 10 and 14 years. The results for the estimated practice population are respectively 17, 9 and 4 serious infections per 1000 children per year.

**Conclusion:**

In contrast to the total incidence of acute infections, serious infections are rare, around 1% per year. Children younger than 4 years old have the highest risk for serious infections, and incidences of some infections are different for boys and girls.

## Background

The incidence of acute infections in children is high. Using data from the English fourth Practice-Based Morbidity Survey, children under 1 year of age reported an average of 3.3 infections in a year, and 1.2 infections in the age group 1–15 years [1]. Acute upper respiratory infection is the dominant diagnosis, which is essentially a harmless, self-limiting disease.

However, some infections can have serious consequences. For example, bacterial meningitis leads to mortality in 5% of the cases and morbidity, such as hearing loss, in 10%–15%[2]. Up to 24% mortality after meningococcal disease, being sepsis or meningitis, has been reported[3]. In developing countries, pneumonia and diarrhoea are two of the leading causes of mortality in children under the age of 5[4]. In the period of 1989–1993, infectious diseases were responsible for 7.5% of all deaths in children under the age of 1, and for 9.3% of all deaths in children between 1 and 14 years old in the Northern region of the UK[5].

Serious infections are usually defined as serious bacterial infections: pneumonia, sepsis, meningitis, pyelonephritis, bacterial gastro-enteritis, osteomyelitis and cellulitis. [6-9]. However, pneumonia and meningitis are often caused by viruses and other viral infections can be serious as well, for example bronchiolitis. In general practice, bacterial aetiology is difficult to establish and the serious nature of the infection is sometimes determined by the need for interventions such as hospitalization or specific treatment. Therefore, in primary care pneumonia, sepsis, meningitis, bacterial gastro-enteritis, pyelonephritis, osteomyelitis and bronchiolitis can be considered serious infections.

The diagnosis of a serious infection is difficult in general practice, as the majority of the infections presented to the general practitioner is not serious and the initial presentation of serious and non-serious infections can be similar. Still, early diagnosis of a serious infection is important to avoid delay in treatment and improve prognosis [10-12].

There is very limited information on the incidence of serious infections in general practice. Most studies were performed in hospital. They have found serious infections such as pneumonia, meningitis, sepsis and bacterial gastroenteritis to be present in up to 25% of febrile infants presenting to the emergency department[13,14]. These results can not be transferred to general practice, because of the large differences between the populations. Other studies in general practice have reported incidences for individual infections [15-17], but differences in health care structure limit the applicability to the Belgian context.

Therefore, we conducted this study to calculate the age-specific incidence of serious infections in children, seen in general practice in Belgium. Additionally, we determined the proportion of serious infections in the entire group of children with an acute infection and we studied seasonal influence.

## Methods

Intego is a morbidity registration network, coordinated by the department of general practice of the Katholieke Universiteit Leuven. 51 general practitioners in 43 practices continuously register all diagnoses and additional data in their electronic medical records. These practices have been selected by the quality of their registration. The information is automatically extracted from the records on a yearly basis and collected in a central database. Diagnoses are classified according to a very detailed diagnostic classification list and converted into ICPC-2 codes[18,19].

As there is free access to all physicians in Belgium, and patients are not forced to register to a practice, it is difficult to establish the practice population. This is particularly important as the incidence of a disease in a certain practice equals all new cases of this disease in that practice, in the nominator, divided by the practice population in the denominator. Until recently, only the group of patients that had consulted their GP at least once in that year has been used as denominator, called the Yearly Contact Group. But, as it was hypothesised that some factors have an influence on the consultation rate of a patient, for example age, gender or social status, it was not possible to extrapolate this Yearly Contact Group to the total practice population, because these influencing factors can be present in certain practices more than in others. Now, data of the Health Insurance Agency have been made available, by which it was possible to estimate a practice population, starting from the Yearly Contact Group and taking age, gender and region into account. This population will be referred to as the Practice Population. A more detailed description of this methodology has been published elsewhere[20].

For children, the difference between the Yearly Contact Group and the Practice Population is particularly large, because some children never consult a general practitioner but only a paediatrician. This makes the estimation of a practice population less appropriate for this age group, because the extrapolation to a larger group that never visits the general practitioner is not very useful.

For this reason, the main outcome measures are presented for both the Yearly Contact Group and the Practice Population. Secondary outcome measures are presented for the Yearly Contact Group only.

Incidences are calculated as the number of events per 1000 patient-years and based upon the data from 1998 to 2002. If a disease occurred more than once during the study period, each occurrence was calculated.

The diagnoses that were analysed are presented in table 1, with their corresponding ICPC-2 code. Serious infections are marked with *.

**Table 1:** ICPC-2 coded diagnoses, included in the study. (* serious infections)

A03 fever
A71 measles
A72 chickenpox
A74 rubella
A75 mononucleosis infectiosa
A76 other viral infection with exanthema
A77 other viral infection
A78 other infection not otherwise specified
D70 gastro-intestinal infection
D71 mumps
D73 supposed infectious gastro-enteritis
H71 otitis media acuta
L70 infection of the limbs*
N71 meningitis/encephalitis + sepsis*
R71 whooping-cough
R72 streptococcal angina
R74 acute infection of the upper respiratory tract
R75 acute/chronic sinusitis
R76 acute tonsillitis
R77 acute laryngitis/tracheitis
R78 acute bronchitis/bronchiolitis
R80 influenza (ex pneumonia)
R81 pneumonia *
R83 other infection of the respiratory tract
U70 pyelonephritis/pyelitis *
U71 acute cystitis

The ICPC-A78 code, other infection not otherwise specified, contains several diagnoses, including sepsis. We extracted the individual diagnoses of sepsis from this group and added them to the code of meningitis, resulting in a combined diagnostic category meningitis/encephalitis/sepsis. On the basis of our data, it was impossible to identify bacterial gastro-intestinal infections within the large group of all gastro-intestinal infections, neither was it possible to separate bronchiolitis from acute bronchitis. These infections could therefore not be included in the selection of serious infections. Serious infections were therefore defined as pneumonia, sepsis, meningitis, osteomyelitis and pyelonephritis.

Analyses were performed with STATA statistical software and Epi-info. 95% confidence intervals are based upon a Poisson distribution if there were 100 cases or less, and on a Normal distribution in all other cases. Differences between age groups were tested with chi^2 ^for trend. A trend over subsequent years was analysed with linear regression analysis, using the year as the independent and yearly incidence as the dependent variable. The seasonal influence was tested in SAS with the combined test for the presence of identifiable seasonality, which is a joint test of both stable and moving seasonality. In the first test, the null-hypothesis is that there is no difference in incidence between seasons. The second test tests whether the seasonal incidence has moved over the last years.

## Results

### Demographics

The Yearly Contact Group consisted in 1998 to 2002 of 45,654 patient years between 0 and 14 years old, of which 47.9% were girls. The total Practice Population is estimated at 60,598 patient years, of which 48.1% were girls.

### Acute infections

The age-specific incidence rates for the total group of the selected acute infections and for the subgroup of serious infections are listed in table 2. Total incidence for all age groups is 1107 acute infections per 1000 patients per year in the Yearly Contact Group or approximately 1.1 infections per child per year. In contrast, the incidence of serious infections is 12.3 per 1000 patients per year, or just over 1 serious infection per 100 children per year. Serious infections account for 1.1% of the acute infections in children selected for this study.

**Table 2:** total incidence rates of all acute infections and serious infections per 1000 patients per year for each age category, between 1998–2002.

	**Denominator**	**0–4 (95% CI)**	**5–9 (95% CI)**	**10–14 (95% CI)**	**Total incidence (95% CI)**
**Acute infections**	Yearly contact group	1731.7 (1708 – 1754)	972.5 (970 – 975)	732.5 (725 – 738)	1106.6 (1097 – 1116)
	Practice population	1383.0 (1365 – 1401)	732.7 (727 – 739)	527.8 (521 – 533)	833.8 (830 – 836)
**Serious infections**	Yearly contact group	21.1 (17.9 – 22.9)	11.7 (9.8 – 13.2)	5.1 (4.0 – 6.3)	12.0 (10.9 – 12.9)
	Practice population	16.8 (14.3 – 18.3)	8.8 (7.4 – 10.0)	3.6 (2.9 – 4.5)	9.3 (8.2 – 9.7)

The incidence of infectious diseases peaks in very young children between 0 and 4 years old, with 1731 infections per 1000 children per year. Incidence drops with increasing age: 972 infections per 1000 children per year in children between 5 and 9 years old, and 732 in children between 10 and 14 years old. The same decline in incidence is observed in the subgroup of serious infections: 21 infections per 1000 children per year in children between 0 and 4 years, 12 in children between 5 and 9 years and 5 in children between 10 and 14 years. These results all apply to the Yearly Contact Group, which is the group of children that consulted their general practitioner at least once during one year. The results for the estimated Practice Population are on average 25% lower than that of the Yearly Contact Group.

When testing the differences between the age groups with chi^2 ^for trend, these are highly significant, both for the total group of infections and for the serious infections, with p-values < 0,001. For serious infections, the odds ratios are 0.56 and 0.25 for children between 5 – 9 years and 10 – 14 years respectively, compared to the group between 0 – 4 years.

**Figure 1:**
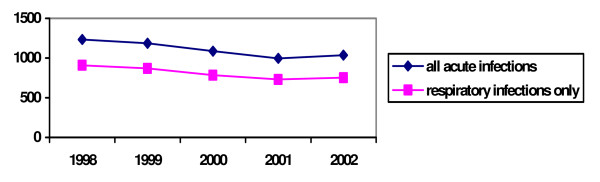
Annual incidence of acute infections per 1000 children of the Yearly Contact Group, from 1998 to 2002.

It has been suggested that there is a decline in incidence of acute infections over recent years[21]. For this reason, we have analysed the annual incidence for each year separately. Although annual incidence of acute infections appears to be declining, there has been a small rise in the annual incidence in 2002 (figure 1). A linear regression analysis shows that there is a marginally significant decline in yearly incidence of 58 acute infections per 1000 children per year from 1998 to 2002 (p = 0.05). A similar decline is to be observed when only respiratory acute infections are considered.

**Figure 2:**
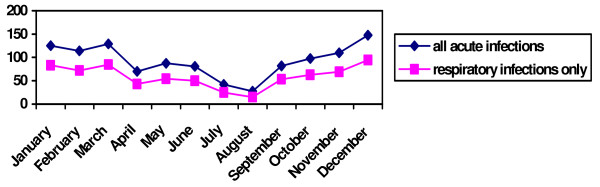
Monthly incidence of acute infections per 1000 children of the Yearly Contact Group, between 1998 – 2002.

The monthly incidence rates are shown in figure 2. Testing for seasonality, there is a higher incidence in the winter for ICPC-codes H71 (otitis media) and the codes R71–R81 (respiratory tract infections) (F < 0.05). These higher incidences are stable over recent years (F > 0.05). Codes A03 and A71–78, being fever and various viral infections, did not show a significant seasonality (F > 0.05).

The incidence per 1000 children per year for each ICPC-2 code is listed in table 3. Considering diagnostic groups separately, the high incidence of acute upper respiratory tract infections (R74) is very prominent: 417 per 1000 patients per year. In children between 0 and 4 years old, this incidence rises to 666 per 1000 per year. When other diagnoses concerning the upper respiratory tract are added, incidence is even higher, being 812 infections per 1000 children of all age groups per year or more than half of all cases. Similar to other diagnostic categories, the youngest children have the highest incidence rate, except for the diagnosis of sinusitis.

**Table 3:** annual incidence rates of acute infections, in the Yearly Contact Group, per 1000 children, from 1998–2002. (* serious infections)

	**0 – 4 years**	**5 – 9 year**	**10 – 14 years**	**Total**
A03 fever	34.9	8.2	2.8	14.0
A71 measles	0.68	0.19	0.13	0.28
A72 chicken pocks	59.7	13.1	1.3	22.4
A74 rubella	0.30	0.63	0.11	0.33
A75 mononucleosis infectiosa	0.68	1.65	2.29	1.60
A76 other viral infection with exanthema	4.5	1.3	0.4	1.9
A77 other viral infection	21.2	11.6	9.7	13.7
A78 other infection nos	8.6	5.1	1.9	4.9
D70 gastro-intestinal infection	10.6	3.1	2.6	5.1
D71 mumps	0.6	0.3	0.5	0.5
D73 supposed infectious gastro-enteritis	130.9	92.1	84.2	100.5
H71 otitis media acuta	226.4	108.8	39.0	117.7
L70 infection of the limbs*	0.45	0.38	0.42	0.42
N71 meningitis/encephalitis + sepsis*	1.4	1.1	0.06	0.7
R71 whooping-cough	0.23	0.00	0.00	0.07
R72 streptococcal angina	0.38	0.44	0.36	0.39
R74 acute infection of the upper respiratory tract	666.5	339.4	292.9	417.8
R75 acute/chronic sinusitis	8.3	18.3	23.1	17.2
R76 acute tonsillitis	87.4	81.7	47.0	70.7
R77 acute laryngitis/tracheitis	84.5	49.7	35.3	54.6
R78 acute bronchitis///bronchiolitis	201.0	89.0	48.5	106.9
R80 influenza (ex pneumonia)	123.7	113.1	118.5	118.1
R81 pneumonia *	18.1	9.9	4.3	10.2
R83 other infection of the respiratory tract	26.7	13.1	10.2	16.0
U70 pyelonephritis/pyelitis *	1.3	0.6	0.4	0.7
U71 acute cystitis	12.8	10.3	6.7	9.7

### Serious infections

The serious infections, for which incidence calculations were possible, are listed in table 4. Pneumonia is the most frequent serious infection in children, accounting for more than four fifth of the total annual incidence.

**Table 4:** annual incidence rates of serious infections, in the Yearly Contact Group, per 1000 children, from 1998–2002.

		**0–4 years (95% CI)**	**5–9 years (95% CI)**	**10–14 years (95% CI)**	**Total (95% CI)**
**Pneumonia**	girls	19.0 (15.8 – 22.7)	10.2 (8.03 – 12.7)	4.0 (2.76 – 5.67)	10.5 (8.78 – 11.5)
	boys	17.3 (14.2 – 22.1)	9.6 (7.64 – 12.0)	4.5 (3.21 – 6.18)	10.0 (8.46 – 11.0)
	total	18.1 (15.8 – 20.5)	9.9 (8.06 – 11.1)	4.3 (3.34 – 5.40)	10.25 (9.18 – 11.0)
**Pyelonephritis/pyelitis**	girls	1.6 (0.75 – 2.91)	0.9 (0.371 – 1.90)	0.5 (0.014 – 1.28)	1.0 (0.59 – 1.47)
	boys	1.0 (0.40 – 2.06)	0.2 (0.03 – 0.88)	0.2 (0.028 – 0.84)	0.4 (0.231 – 0.83)
	total	1.3 (0.74 – 2.04)	0.6 (0.26 – 1.08)	0.4 (0.13 – 0.79)	0.7 (0.48 – 0.99)
**Meningitis/Sepsis**	girls	1.4 (0.65 – 2.71)	0.5 (0.015 – 1.35)	-	0.6 (0.32 – 1.02)
	boys	1.3 (0.59 – 2.45)	1.3 (0.50 – 2.09)	0.1 (0.03 – 6.46)	0.8 (0.45 – 1.19)
	total	1.4 (0.80 – 2.14)	1.1 (0.44 – 1.41)	0.06 (0.02 – 3.36)	0.7 (0.48 – 0.99)
**Infection of the limbs**	girls	0.2 (0.004 – 0.88)	0.4 (0.082 – 1.16)	0.4 (0.078 – 1.10)	0.3 (0.12 – 0.66)
	boys	0.7 (0.23 – 1.67)	0.4 (0.076 – 1.07)	0.5 (0.13 – 1.19)	0.5 (0.26 – 0.88)
	total	0.5 (0.17 – 0.98)	0.4 (0.14 – 0.83)	0.4 (0.17 – 0.87)	0.4 (0.25 – 0.65)
**Total**	girls	21.7 (17.2 – 26.2)	12.0 (9.67 – 14.8)	4.9 (3.48 – 6.69)	12.2 (10.5 – 13.4)
	boys	20.3 (16.9 – 23.6)	11.4 (9.17 – 13.9)	5.2 (3.81 – 7.00)	11.7 (10.4 – 13.2)
	total	21.1 (17.9 – 22.9)	11.7 (9.81 – 13.2)	5.1 (4.04 – 6.27)	12.0 (10.9 – 12.9)

Except for infection of the limbs, all infections are most frequent in the youngest children.

**Figure 3:**
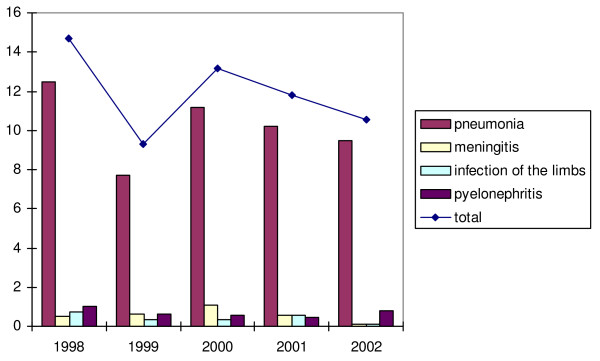
Annual incidence of the serious infections per 1000 children of the Yearly Contact Group, from 1998 to 2002.

The evolution of the yearly incidence of each serious infection is shown in figure 3. It is influenced mainly by the incidence of pneumonia. The decline of the annual incidence of the group of serious infections in 1999 can therefore be explained by the decline of annual incidence of pneumonia. Ignoring this drop, there could be a decline in annual incidence, parallel to that of the total group of acute infections. However, a linear regression analysis based on all subsequent years does not show any significant decline in yearly incidence (p = 0.49).

There is a peak of the annual incidence of meningitis in 2000, followed by a steep decline in the two years afterwards (figure 3). Pyelonephritis and infections of the limbs do not show any particular trend.

**Figure 4:**
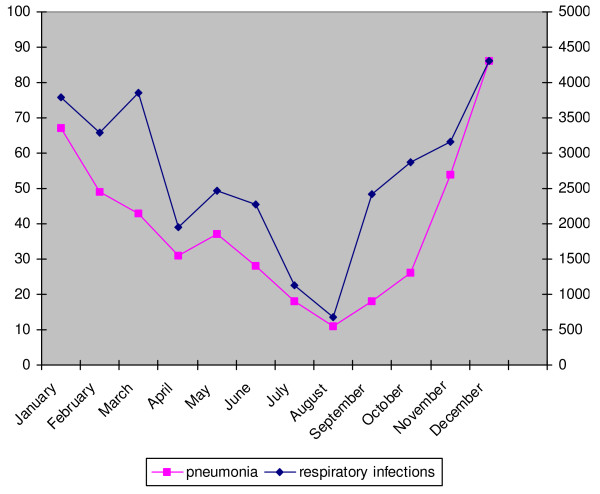
Absolute number of cases of pneumonia and respiratory infections monthly, in the Yearly Contact Group, between 1998–2002*. *The axis for pneumonia is situated on the left of the figure, the axis for respiratory infections on the right.

The absolute numbers of cases of pneumonia and all respiratory infections in each month are given in Figure 4.

Similar as all respiratory infections, the incidence rates of pneumonia are significantly higher during the winter (figure 4) (F < 0.05). This seasonal trend is stable over the last years (F > 0.05).

The incidence rates of meningitis do not show a similar variation over the different months as pneumonia. The combined seasonality test showed a higher incidence in the fall, but this was not stable over the last years. Pyelonephritis shows a higher incidence in spring and summer, which was stable over the last years (F < 0.05 and >0.05 respectively).

## Discussion

The incidence of acute infections in children is high. For every 1000 children between 0 and 14 years, over 1100 acute infections are presented to the general practitioner in Belgium every year. The vast majority of these infections are self-limiting upper respiratory tract infections.

In contrast, serious infections are relatively rare: only 12.0 serious infections per year in every 1000 children between 0 and 14 years old. This is an annual incidence of little more than 1%, and accounts for 1.1% of the selected acute infections in this age group. As we were unable to separate bacterial gastro-intestinal infections or bronchiolitis, the true incidence of serious infections is higher, especially in the youngest age group.

Incidence of acute infections seems to be declining over the last five years, which has been described before[21]. For serious infections, this evolution is less clear.

In 2000, there was an outbreak of meningococcal C infections in Western Europe, leading to widespread vaccination programmes in children. This is reflected in our results.

Incidences are highest in the youngest children between 0 and 4 years old. This is true for all acute infections (1732 per 1000 patients per year in the age group 0 to 4 years compared to 1107 in all children, which is an excess of 64%), as well as for serious infections only (21.0 between 0 and 4 years compared to 12.3 in all children, which is an excess of 75%). This peak incidence during the first years of life has been described before [22-25]. The only exception in our data was the incidence of sinusitis, where incidence rises with increasing age. This can be explained by the fact that sinuses are not yet well developed in very young children.

Incidence rates vary during the year, with all acute infections and pneumonia being more frequent in the winter months. For other serious infections, no particular pattern could be observed, although the peak of meningitis in the fall and that of pyelonephritis in the warmer season need to be examined further.

Our results were subsequently compared to other morbidity registrations. Data from three neighbouring registrations were available, being the Transition project^21 ^and the Continuous Morbidity Registration (CMR), both from the Netherlands, and the Weekly Returns Service, from the UK[21]. Data were not available from all networks for every diagnosis, except for pneumonia, but the incidences of the Intego network, the Transition project and the Weekly Returns Service are within the same range.

The CMR reports higher incidences for pneumonia as the other three registrations: in children aged 0 – 4 years, the yearly incidence is 27–30 per 1000 patients, as compared to 18.3 in the Transition project, 18.1 in our present study based on Intego and 15.7 in the Weekly Returns Service (the latter is based on children from 1–4 years old). Another outlier is the very high incidence of meningitis in children under the age of one, reported by the UK Weekly Returns Service: 9.4 infections per 1000 children per year, as compared to a yearly incidence of 1.4 per 1000 in Intego and 1.3 per 1000 in the Transition project, both based however on children from 0 to 4 years old.

But, any comparison between the different morbidity registrations is difficult. Firstly, populations are different. For example, the health care structure in the UK is totally different from that in Belgium, as there is free access to specialist care in Belgium. In general, GPs are informed about all diseases of their patients including those who went directly to a specialist. However, for young children who only have been seen by a paediatrician and not by a GP, this may not always be the case. Therefore, we used the Yearly Contact Group instead of the Practice Population for most analyses.

Although the method for estimating the Practice Population is robust as it takes several influencing factors for consultation rate into account, the true Practice Population remains undefined[26]. Even in those countries with a fixed registration to a particular GP, the true Practice Population is constantly changing. In the Belgian context, uncertainty on the true practice population is even more important. Therefore, calculations using the Practice Population should be considered with caution.

Because of free access to specialist care, our results should be interpreted as the incidence rates of infections in general practice only. Any decline in incidence could reflect a true decline or a shift in consultation practices. This 'competition' between general practice and paediatricians explains the lower incidence of meningitis in our data as compared to other morbidity registrations, because parents with very young children in Belgium tend to consult the paediatrician. The influence of differences in health care structure could be the scope of further research.

The period of registration is another influencing factor, as incidences of acute infections seem to be decreasing during the last decade[21,27].

Finally, diagnoses are defined differently. The Transition project uses the ICPC, the Weekly Returns Service uses the ICD coding system, the Continuous Morbidity Registration an adapted version of the E list and the Intego network an extensive own list, but we recoded all diagnoses according to the ICPC-2 for analysis. For example, the Weekly Returns Service reported a higher incidence of meningitis of 9.4 infections per 1000 children less than one year old, which can be explained by the fact that this database contains unconfirmed cases.

## Conclusion

In contrast to the total incidence of the selected acute infections, serious infections are rare in general practice, around 1% per year. Children younger than 4 years old have the highest risk for serious infections, and incidence of pneumonia peaks in the winter months.

## Competing interests

The author(s) declare that they have no competing interests.

## Authors' contributions

AVDB carried out the analysis and drafted the manuscript. SB provided the data and commented on the manuscript. BA participated in the study design and commented on the manuscript. CT participated in the analysis and provided statistical support. FB conceived of the study, and participated in its design and coordination and helped to draft the manuscript. All authors read and approved the final manuscript.

## Pre-publication history

The pre-publication history for this paper can be accessed here:


